# Safety and effectiveness of sleeve gastrectomy versus gastric bypass: one-year results of Tehran Obesity Treatment Study (TOTS)

**Published:** 2016-12

**Authors:** Maryam Barzin, Alireza Khalaj, Mohammad Ali Motamedi, Pravin Shapoori, Fereidoun Azizi, Farhad Hosseinpanah

**Affiliations:** 1 *Obesity Research Center, Research Institute for Endocrine Sciences, Shahid Beheshti University of Medical Sciences, Tehran, Iran *; 2 *Faculty of Medicine, Shahed University, Tehran, Iran*; 3 *Minimally Invasive Surgery Research Center, Rasoul-e-Akram Hospital, Iran University of Medical Sciences, Tehran, Iran *; 4 *Endocrine Research Center, Research Institute for Endocrine Sciences, Shahid Beheshti University of Medical Sciences, Tehran, Iran*

**Keywords:** Morbid obesity, Bariatric surgery, Laparoscopy, Sleeve gastrectomy, Gastric bypass

## Abstract

**Aim:**

We aimed to compare the effectiveness and safety of sleeve gastrectomy versus gastric bypass at one-year.

**Background:**

Roux-en-Y gastric bypass (RYGB) and sleeve gastrectomy (SG) are the two most commonly performed bariatric procedures worldwide.

**Methods:**

Patients from a prospectively collected database who presented to a specialized bariatric center and underwent a primary bariatric procedure between March 2013 and April 2015 were included and compared regarding major and minor complication rates and weight loss parameters at 6 and 12 months.

**Results:**

A total of 513 patients with a mean age of 37.5±12.5, 82.6 % female and mean body mass index (BMI) of 44.1±6.3 kg/m2 were included in our analysis: 73.3% underwent SG and 26.7% underwent RYGB. Major and minor complication rates were 7.1 and 2.6% for SG vs. 9.5 and 2.2% for the RYGB, respectively (*P*=NS). The operative and anesthesia time in SG patients were significantly shorter than in RYGB patients (P<0.001). SG and RYBG patients achieved similar excess weight loss at one year (75.4±20.5% vs. 71.8±26.3%, respectively, *P*=NS). Baseline BMI was the only predictive factor for weight loss at one year (OR: 0.901, CI: 0.827-0.982, *P*<0.017).

**Conclusion:**

RYGB and SG both showed similar one-year safety and effectiveness. Long-term studies are needed to complement these findings.

## Introduction

Bariatric surgery has proved to be the most effective treatment for morbid obesity and its comorbidities, and is increasingly performed worldwide (1). During the past several years, various new techniques have been introduced and incorporated into practice. Introduced initially as part of a two-stage bariatric procedure (2), sleeve gastrectomy (SG) has shown promising outcomes as a stand-alone operation and is today among the most preferred techniques in the US, surpassing laparoscopic Roux-en-Y gastric bypass (RYGB) in number (3, 4). Nonetheless, RYGB is still more widely accepted as the gold standard in many centers.

Regarding the effectiveness and safety, studies directly comparing these two techniques have shown inconsistent results. The majority favor superiority of RYGB in excess weight loss (EWL) terms but at the expense of higher complication rates, as was shown in a recent meta-analysis (5), which concluded that RYGB is superior in terms of EWL in the long term (>1.5 years) but not in terms of post- operative adverse events.

Considering this and the scarcity of data in the Middle East region, we decided to undertake a study of these popular bariatric techniques, evaluating their safety and effectiveness.

Here we report the one-year outcomes of the Tehran Obesity Treatment Study (TOTS) study.

## Materials and Methods

**Design and setting**

The TOTS is an ongoing prospective study commencing on March 2013, evaluating morbidly obese patients for bariatric surgery. Surgery takes place in three university hospitals in Tehran. Numerous pre-, intra- and postoperative parameters, as well as short and long-term follow-up data are gathered by trained personnel using validated protocols and entered into the specifically-designed computer database. More details of this study is described elsewhere (6).

**Participants**

Morbidly obese patients who met the study criteria between March 2013 and April 2015 were selected to enter the study after providing written informed consent. Participation of each patient was discussed in multiple counseling sessions with the presence of a multi-disciplinary treatment team (surgeon, psychiatrist, nutritionist, and obesity expert). Each participant then underwent multiple medical consultations to evaluate obesity-related comorbidities, suitability for surgery and the choice of surgical technique.

**Procedures**

Participants in the TOTS underwent SG or RYGB. A single surgical team (KA and SP, two fellowship bariatric surgeons) performed all operations with a standard 5-port laparoscopic approach under general anesthesia. SG was performed over a 36-F bougie and reinforced with running sutures. RYGB was performed with construction of a vertical pouch of stomach and anastomosis to an antecolic 150 cm roux limb of jejunum and a side-to-side jejunojejunostomy with a 50 cm biliopancreatic limb. A methylene blue or air test was performed to check for any leaks, and a closed suction drain was placed based on the surgeon’s discretion.

**Variables and outcomes**

The study variables included demographics, past and current medical history, medications, social history (alcohol use, smoking, drug abuse), anthropometric measurements, body composition, and obesity-related comorbidities. Intraoperative variables include anesthesia time (from the time of anesthesia induction to recovery), operative time (from first incision to the last stitch), and conversion-to- open rate. Outcome categorization and reporting is according to the American Society of Metabolic and Bariatric Surgery (ASMBS) outcome reporting guidelines (7). Major complications were defined as those requiring return of the patient to the operating room for reintervention or reoperation, prolonged hospital stay beyond 7 days, and those requiring anticoagulant administration. All other complications were regarded as minor. For weight loss analysis, ideal weight was defined by the weight corresponding to a BMI of 25 kg/m2. Our primary endpoints for this study were early (<30 days) and late (> 30 days), major and minor complication rates, length of hospital stay, operative times, and weight loss parameters at 1, 3, 6, and 12 months postoperatively. Secondary outcome was defined as any correlations between the baseline characteristics and weight loss at one year.

**Statistical analysis**

Data were analyzed using IBM SPSS for Windows (version 20). The normality of continuous variables was checked using the Kolmogorov-Smirnov test and presented as mean ± standard deviation or median (IQ 25-75) where applicable. Categorical variables were presented as percentages. Differences between groups in normally distributed continuous variables were assessed using the independent samples t-test, and categorical variables were compared using the χ2 and the Fisher’s exact test. Repeated-measure analyses of variance were performed to test whether time trends differ between the two groups, and whether there is a time effect within each treatment group. Multivariate logistic regression methods were used to estimate the adjusted odds ratios and 95% confidence intervals of excess weight loss >50% vs. <50% after adjustment for baseline covariates consisting of age (year), sex (ref: male), BMI (kg/ m2), type of procedure (ref: SG), hypertension, and diabetes mellitus.

*P *value of less than 0.05 was considered significant.

**Ethical approval**

All procedures performed were in accordance with the 1964 Helsinki declaration and its later amendments or comparable ethical standards. The study has been reviewed and approved by the Human Research Review Committee of the Endocrine Research Center, Shahid Beheshti University of Medical Sciences (No. 2ECRIES 93/03/13). All participants gave their informed consent prior to their inclusion in the study.

## Results

**Figure 1:**
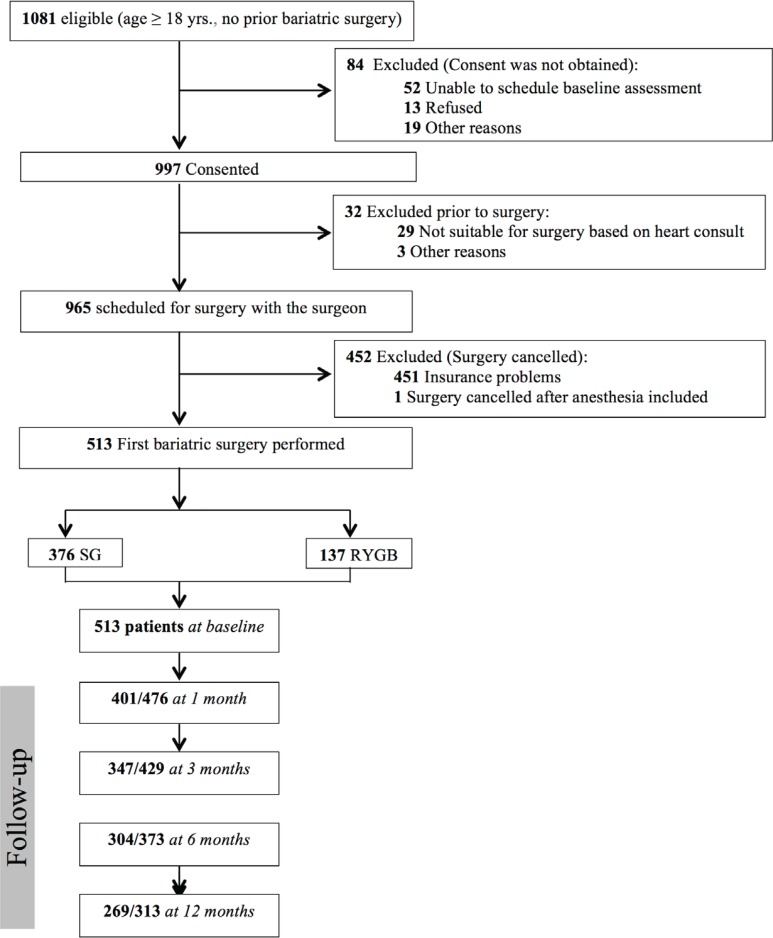
Recruitment flow chart of the study participants. RYGB, Roux-en-Y gastric bypass; SG, sleeve gastrectomy

Recruitment flow is presented in figure 1. Of the 1080 patients potentially eligible for the study, 513 participants who had their first bariatric procedure performed during the study period were included; 376 (73.3%) underwent SG and 137 (26.7%) underwent RYGB.

Baseline characteristics of the participants are presented in table 1. The mean age of the participants was 37.5 ±12.5 years (range 18-69 years). The majority of participants were female (82.6%), and the RYGB group had a higher proportion of women compared to the SG group (86.1% vs. 76.6%, *P<*0.001). Overall, among the common obesity-related comorbidities, hypertension was seen in 18.9% of all patients, followed by diabetes mellitus (16.6%) and hyperlipidemia (9%). The SG group had a lower percentage of diabetes mellitus (14.9 vs. 23.6%, *P*=0.037) and dyslipidemia (6.1 vs. 17.2%, P< 0.001). There were no significant differences in other baseline characteristics between the two groups.

**Table 1:** Baseline characteristics and preoperative anthropometrics of the participants by procedure †

Characteristic	SG N=376 (73.3%)	RYGB N=137 (26.7%)	P-value
Age, year	37.7 ±12.5	37.0 ±12.8	NS
Age category, n (%)			
<40 y	220 (58.5)	80 (58.4)	NS
≥40 y Sex, n (%)	156 (41.5)	57 (41.6)	NS
Age, year			
Female	288 (73.4)	118 (92.9)*	<0.001
Male	88 (23.4)	19 (7.1)*	<0.001
Smoke category, n (%)			
Non-smoker	275 (75.8)	101 (73.7)	NS
Smoker	25 (7.6)	10 (7.3)	NS
Weight, kg	119.7 ±22.1	116.8 ±17.8	NS
Height, cm	163.9 ±9	162.6 ±6.9	NS
BMI, kg/m2	44.5 ±6.1	44.1 ±5.2	NS
BMI category, n (%)			
<50 kg/m2	319 (84.9)	119 (86.9)	NS
≥50 kg/m2	57 (15.1)	18 (13.1)	NS
Diab`etes mellitus, n (%)	54 (14.9%)	31 (23.6%)*	0.037
Hypertension, n (%)	74 (20.4%)	23 (17.3%)	NS
Dyslipidemia, n (%)	24 (6.1%)	22 (17.2%)*	<0.001
Sleep apnea, n (%)	20 (5.5%)	6 (4.7%)	NS

SG, sleeve gastrectomy; RYGB, Roux-en-Y gastric bypass; BMI, body mass index; NS, not significant.

† Values are mean ±SD

* *P *< 0.05, compared with SG

**Table 2.** Early and late major and minor complications following surgery up to one-year follow-up

Variable	SG N=376(73.3%)	RYGB N=137 (26.7%)	*P *value†
Length of hospital stay, day, mean ± SD (range)	2.8 ± 3.1 (2-18)	2.4 ± 2.6 (2-13)	0.333
Surgery time, min, mean ± SD	63.9 ±17.9	89.4±34.1	<0.001
Anesthesia time, min, mean ±SD	128.4 ±24.9	153.2 ±37.3	<0.001
Conversion to open, n (%)	1 (0.03)	0	NS
**Major** ** Complications, n (%)**			
**Early** ** (30-day)**			
Death	0	0	-
Return to operating room	8 (2.1%)	6 (4.3%)	NS
Port-site bleeding	4 (1%)	2 (1.4%)	NS
Abscess/Infection	3 (0.7%)	1 (0.7%)	NS
Anastomotic stricture	0	1 (0.7%)	NS
Staple line leak	1	0	NS
Marginal ulcer perforation	0	1 (0.7%)	NS
Internal hernia	0	1 (0.7%)	NS
Deep venous thrombosis	0	1 (0.7%)	NS
Pneumonia	1 (0.2%)	0	NS
Bleeding requiring transfusion	7 (1.8%)	4 (2.9%)	NS
Prolonged hospitalization (>7 d)	16 (4.2%)	6 (4.3%)	NS
*Subtotal* * n (%)*	16 (4.2%)	11(8.0%)	0.333
**Late (>30 ** **days up to one ** **year)**			
Death††	2	0	NS
Symptomatic cholelithiasis /Cholecystectomy	5	1	NS
Nephrolithiasis	2	1	NS
Incisional hernia (diagnosed during follow-up)	2	0	NS
*Subtotal,* * n (%)*	11 (2.9%)	2 (1.5%)	0.064
*T* *otal* * major * *complications,* * n (%)*	27 (7.1%)	13 (9.5%)	0.458
**Minor** ** Complications**			
Dehydration requiring inpatient intravenous therapy	2	1	NS
Marginal ulcer diagnosed and treated with upper endoscopy	0	1	NS
Anastomotic stricture requiring endoscopic dilation	2	0	NS
Nausea and vomiting requiring intravenous fluids but not TPN	3	0	NS
Urinary tract infection managed with antibiotics	3	1	NS
*Subtotal* * n (%)*	10 (2.6%)	3 (2.2%)	NS

SG, sleeve gastrectomy; RYGB, Roux-en-Y gastric bypass; TPN, total parenteral nutrition;

† For comparison between the two groups. Student t-test, Chi-squared or Fisher’s Exact tests were used based on variable type and the value of each cell,when appropriate.

†† Two cases of advanced cancer diagnosed during follow-up.

The mean length of hospital stay was 2.8 and 2.4 days in the SG and RYGB groups, respectively (*P*=0.333). The mean operative and anesthesia times were significantly shorter in the SG group compared with the RYGB group (63.9 ±17.9 and 128.4 ±24.9 vs. 89.4 ±34.1 and 153.2 ±37.3 minutes, respectively, P<0.001). Table 2 presents the postoperative complications. There were two late mortalities in the SG group due to cancer, which was diagnosed during the post-op visits. One of the cases in the SG group was converted to an open procedure because of extensive intra-abdominal adhesions. Fourteen patients returned to the operating room: 8 (2.1%) in the SG group and 6 (4.3%) in RYGB group (*P *=0.217).

**Figure 2:**
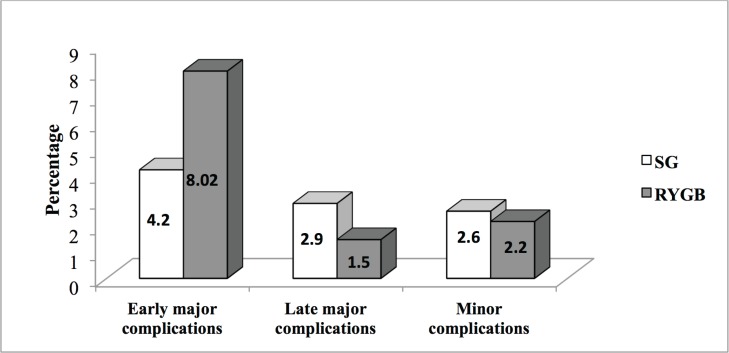
Early (<30 days) and late (>30 days to 1 year) major and minor complication rates in the sleeve Gastrectomy (SG) and Roux-en-Y Gastric Bypass (RYGB) groups

**Table 3:** Weight loss parameters in the two groups at 1, 3, 6, and 12 months †, ‡, §

		BMI	ΔBMI	%EWL	%EBMIL	%TWL
*SG*	*1 month*	38.9 (36.1_ 43.3)	-4.3 (-3.5_ -5.2)	22.9 (19.2_ 27.6)	22.8 (19.0_27.7)	9.8 (8.1_ 11.4)
	*3 months*	35.8 (32.9_ 39.6)	-8.2 (-6.8_ -9.5)	42.6 (35.1_ 52.0)	42.3 (34.6_52.0)	18.5 (15.6_ 20.9)
	*6 months*	32.8 (30.0_ 36.4)	-11.4 (-9.3_ -13.5)	57.5 (49.2_ 71.0)	57.5 (49.5_70.5)	25.0 (21.6- 29.8)
	*12 months*	29.8 (26.4_ 33.3)	-14.1 (-11.5_ -17.0)	80.7 (58.7_ 88.5)	75.5 (60.5_90.1)	32.6 (26.4_ 37.4)
*RYGB*	*1 month*	39.6 (36.4_ 42.7)	-4.3 (-3.3_ -5.3)	22.2 (19.5_ 27.0)	22.1 (18.7_27.8)	9.7 (7.7_ 11.8)
	*3 months*	35.8 (32.8_ 38.5)	-7.8 (-6.7_ -9.5)	42.3 (35.3_ 51.2)	43.1 (36.4_49.6)	18.1 (15.3_ 20.4)
	*6 months*	32.4 (29.3_ 36.0)	-11.5 (-9.9_ -12.8)	60.2 (50.4_ 74.5)	60.2 (50.2_74.4)	26.1 (22.2_ 30.1)
	*12 months*	30.3 (26.8_ 34.9)	-13.5 (-10.3_ -16.2)	71.8 (57.5_ 89.7)	70.8 (53.1_88.9)	30.7 (24.3_ 36.7)

SG, laparoscopic sleeve gastrectomy; RYGB, laparoscopic Roux-en-Y gastric bypass; BMI, body mass index; ΔBMI, change in BMI units; EWL, excess weight loss; EBMIL, excess BMI unit loss; TWL, total weight loss;

† Data was available in 401/476 (84.2%) at 1 month, in 347/429 (80.8%) at 3 months, in 304/373 (81.5%) at 6 months, and in 269/313 (85.9%) at 12 months postoperatively.

‡ Values are median (25-75 IQ)

§ *P *value not significant after adjustment for sex, diabetes mellitus, and dyslipidemia at any time point

The rate of early major complications was slightly higher in the RYGB group (8 vs. 4.2%, *P*=0.333), and the rate of late major complications slightly higher in the SG group (2.9 vs. 1.5%, *P*=0.064), neither reaching statistical significance. Minor complication rates were also similar between the two groups (2.6% and 2.2% in the SG and RYGB groups, respectively, *P*>0.05, figure 2).

The weight loss analysis was based on 1-year follow-up data, which was available in 401/476 (84.2%) at 1 month, in 347/429 (80.8%) at 3 months, in 304/373 (81.5%) at 6 months, and in 269/313 (85.9%) at 1 year. Lost to follow-up rate difference was not significant between the two groups. The mean postoperative EWL percentages at 1, 3, 6, and 12 months were 22.9%, 42.6%, 57.5%, and 80.7% in the SG group and 22.2%, 42.3%, 60.2%, and 71.8% in the RYGB group, respectively (*P*=not significant at any time point). Furthermore, there were no significant differences in BMI unit loss (ΔBMI), excess BMI loss (EBMIL), and total weight loss (TWL) between the two groups. These differences in weight loss were not significant either after adjustment for sex, diabetes mellitus, and dyslipidemia. Detailed data regarding these parameters are presented in table 3.

Multivariate analysis with adjustment for possible variables revealed a significant correlation between preoperative BMI and weight loss, such that patients with lower preoperative BMI achieved higher EWL≥ 50% at one year (OR: 0.901; 95%CI: 0.827-0.982, *P*=0.017).

## Discussion

We found that the one-year weight loss results of SG are comparable to RYGB while having a similar early and late morbidity rate. Although RYGB is generally considered the gold standard bariatric procedure worldwide, SG is rapidly becoming a popular and acceptable procedure. The choice between these two procedures often poses a challenge even for the experts in this field.

Complication rates are of much debate in different studies, with variable rates for each procedure. Many authors have shown higher morbidity rates for RYGB compared with SG (5, 8-10), such as a recent meta-analysis by Zhang et al (5). However, we found no significant difference with regard to major and minor complications. This is in accordance with some studies showing similar results (11, 12). This could be partly explained by the absence of a common reporting standard between studies, as well as variations in the surgical technique or expertise between centers.

The SG has been shown to be shorter in operative times in multiple studies (8, 10, 12, 13), as well as in our own. This can have useful implications in terms of cost-effectiveness, especially in centers with long waiting lists. However, its effect on length of hospital stay, complication rate, and long- term follow up is less clear and not sufficiently investigated. Some studies have shown shorter hospital stay for SG as well (10), although they were similar in our study. Further studies comparing efficacy of various bariatric procedures may reveal a superiority of one over the other in the future. Weight loss as the main outcome of bariatric surgery has been widely investigated. Studies have basically shown different patterns of weight loss when comparing SG with RYGB. There are data showing better results for RYGB than SG in the short, (9, 14-16) mid, (5, 11) or long-term (5). Conversely, some studies reported similar (1, 14) or even better weight loss for SG than RYGB in the short and mid- term (1, 12, 16-18). We found slightly better results for SG compared to RYGB at one year. There is no clear explanation for this discrepancy, but the difference in technical details (e.g. length of jejunal limbs, extent of fundal resection, etc.) could be responsible. This variability in technical detail has been shown to be significant in one study (19) and not in another (20). One can also speculate a relationship between this issue and the underlying mechanism of weight loss, which is yet not fully understood. A large remaining fundus can be linked to secretion of ghrelin, affecting the weight loss results (21). However, this report presents the one-year outcomes, and a difference may become evident over the long term.

We found a relationship between preoperative BMI as a negative predictor for weight loss, but not other factors. This relationship has been frequently reported in studies showing super-obese patients achieving lower EWL. Other factors mentioned in the literature include participating in social support groups, preoperative weight loss, personality disorders and binge eating as negative predictors (22-24).

Although we found similar outcomes for SG and RYGB, the authors believe that a major advantage for SG is the preservation of endoscopic access to the remnant stomach, which can improve screening and diagnosis of gastric cancer. This particularly comes into play in regions with a high incidence of gastric cancer, like our country, where it is the most common cause of cancer deaths in both sexes (25, 26). Some limitations in the present study must be mentioned. First, this study was not a randomized trial, and allocation of patients into two groups was finally based on their preference if no contraindications existed regarding each procedure. Second, although resolution of comorbidities is a major outcome of bariatric surgery, we did not analyze this data since it was beyond the scope of this report. However as a major strength, the standardized protocol for assessment, surgery, and follow- up of patients enables readers and future investigators to explicitly and clearly compare our results with their own.

In conclusion, this study demonstrates that the safety and effectiveness of SG is comparable to RYGB at one-year follow- up. Considering the easier and faster technique of SG and the advantage of preservation of endoscopic access to the stomach and high prevalence of gastric cancer in our population, this procedure could particularly be a suitable choice for surgeons in this region. Long-term randomized trials need further investigation to compare the effectiveness and safety of SG with other procedures.
